# When is AI “just another innovation”? A comparative conceptual analysis of artificial intelligence and evidence-based practice implementation

**DOI:** 10.1186/s43058-026-01103-w

**Published:** 2026-09-10

**Authors:** Per Nilsen, Kathrine Hald, Margit Neher

**Affiliations:** 1 Department of Health, Medicine and Caring Sciences, Division of Public Health, Faculty of Health Sciences, Linköping University, Linköping, Sweden; 2 Department of Clinical Medicine, Sweden Center for General Practice, Aalborg University, Aalborg, Denmark; 3 Danish Center for Health Services Research, Department of Clinical Medicine, Aalborg University, Aalborg, Denmark; 4 Australian Institute of Health Innovation, Macquarie University, Sydney, Australia; 5 School of Health and Welfare, Halmstad University, Box 823, Halmstad, SE 301 18 Sweden

**Keywords:** Implementation, Implementation science, Debate, Artificial intelligence, Evidence-based practice, Intervention, Innovation, Sociotechnical systems, Lifecycle governance

## Abstract

**Background:**

Implementation science frameworks, such as the Consolidated Framework for Implementation Research (CFIR), are increasingly used to guide the implementation of artificial intelligence (AI) in healthcare. This assumes that AI systems can be understood using frameworks developed primarily for evidence-based practices (EBPs). However, AI systems vary substantially in their technical architecture, degree of autonomy, dependence on local data, regulatory status and capacity for change after deployment.

**Aim:**

To examine how different types of AI systems align with, extend or challenge core assumptions in implementation science, using CFIR as a diagnostic lens.

**Method:**

We conducted a comparative conceptual analysis of foundational implementation science literature, literature on complex interventions and technology-enabled care, and empirical and conceptual studies on AI implementation in healthcare. CFIR was used as an analytic lens to identify points of fit and tension across its five domains. The analysis was not designed as a systematic or scoping review, but as a theoretically informed comparison of recurring concepts and assumptions across bodies of literature.

**Results:**

AI systems differ substantially in their implementation implications. Fixed or locked AI tools may resemble conventional digital interventions, whereas adaptive, data-dependent and generative AI systems raise more substantial challenges. Our analysis suggests that, across CFIR domains, adaptive and generative AI systems foreground issues such as opacity, probabilistic outputs, dependence on local data ecosystems, performance drift, vendor-mediated updating, regulatory uncertainty, professional identity tensions and the need for calibrated trust. These issues can often be mapped to existing frameworks, but in some cases they challenge assumptions of intervention stability, boundedness and evidentiary closure.

**Conclusions:**

AI should not be treated as a homogeneous implementation object. Existing implementation science frameworks remain analytically valuable, but require refinement when applied to AI systems whose behavior depends on changing data, infrastructure, governance and use contexts. Implementation of such systems is better conceptualized as lifecycle stewardship than as a bounded rollout.

**Contributions to the literature**

- The paper distinguishes between fixed, adaptive and generative AI systems and shows that their implementation implications differ substantially.
- Using CFIR as a diagnostic lens, the paper identifies where AI-related issues can be accommodated within existing implementation frameworks and where they challenge assumptions of stability, boundedness and evidentiary closure.
- The paper situates AI implementation within existing debates on complex interventions, fidelity versus adaptation, sociotechnical systems.
- The paper presents a nuanced discussion of the distinctive complexities in the implementation of some AI systems, while avoiding the claim that all AI implementation challenges are wholly novel.
- We propose theoretical refinements for AI implementation, including lifecycle stewardship, data ecosystem governance, vendor governance, calibrated trust and continuous ethical oversight, and argue that model drift, calibration, subgroup performance and fairness should be monitored alongside implementation outcomes as part of lifecycle evaluation.

## Introduction

Artificial intelligence (AI) is widely portrayed as a transformative force in healthcare and public services, promising enhanced decision-making, improved efficiency, and better outcomes. Yet, despite rapid advances in algorithmic capability, many AI initiatives struggle to move beyond pilot phases or fail to achieve sustained routine use [1–4]. This implementation gap has spurred efforts to mobilize implementation science to guide AI deployment. In particular, determinant frameworks and process models originally developed to support the uptake of evidence-based practices (EBPs) [5] have been proposed as organizing structures for AI implementation [6–9].

EBPs are commonly understood as clinical, organizational or public health practices whose use is supported by systematically produced research evidence, often integrated with professional expertise and patient preferences [10]. Examples include a guideline-recommended medication protocol, a sepsis care bundle, a smoking cessation intervention, a falls-prevention programme or a standardized screening pathway. These interventions vary in complexity and adaptability, but their evidentiary basis, intended users and core functions can usually be specified before implementation. Within implementation science, EBPs have served as a central object of study: the field has developed theories, models and frameworks to understand how such practices are adopted, implemented, sustained, adapted and scaled in real-world settings.

At first glance, applying implementation science to AI appears well founded. Implementation science offers structured determinant frameworks [5], such as the Consolidated Framework for Implementation Research (CFIR) [11, 12], which enable the systematic identification of barriers and facilitators across multiple levels of context. In addition, the field provides process models [5] that guide the implementation of EBPs through clearly defined stages of activities for successful implementation. If AI is “just another innovation,” then existing implementation frameworks and models should suffice. However, this assumption warrants closer scrutiny because many AI systems, particularly adaptive, data-dependent and generative systems, may differ from traditional EBPs in important ways.

In this paper, we do not treat AI as a single homogeneous class of intervention. AI systems vary substantially in their technical architecture, degree of autonomy, update mechanisms, dependence on local data, regulatory status and clinical role. A locked or fixed AI tool, such as a pre-approved image-classification algorithm whose model weights do not change after deployment, may resemble a conventional technology-enabled intervention in many respects. By contrast, adaptive machine-learning systems, continuously updated models and generative AI systems may change in performance, outputs or use implications over time. Generative AI and foundation-model-based systems introduce additional implementation issues because they can produce open-ended outputs, interact conversationally with users, and be repurposed across tasks beyond their original intended use [13, 14].

Examples of AI systems in healthcare include a locked radiology algorithm for detecting pulmonary nodules, a machine-learning risk prediction model trained on historical electronic health record data, an adaptive clinical decision support system that is periodically recalibrated as new data accumulate, and a generative AI tool used to summarize clinical notes or support patient communication. These systems differ substantially in their implementation implications. The more open-ended, adaptive and data-dependent the system, the more strongly it challenges assumptions of stability and boundedness.

We also recognize that traditional EBPs are not always simple, fixed or context-independent. Implementation science has long debated fidelity versus adaptation, intervention form versus function, and the difficulty of distinguishing an intervention from the system in which it is implemented [15–19]. Complex interventions may evolve through local tailoring, professional interpretation and organizational learning. Consequently, the distinction between EBPs and AI should not be understood as a binary contrast between static and dynamic objects. Rather, AI systems sit on a continuum of complexity. What makes some AI systems distinctive is that their outputs and performance may change not only because humans adapt them in practice, but also because data distributions shift, models are updated, interfaces change, vendors modify systems, or generative systems produce variable outputs across similar prompts or contexts.

Using the updated CFIR [12] as an analytic lens, this paper identifies key areas where AI implementation differs from, overlaps with, and extends the implementation of EBPs. We do not argue that all AI systems are fundamentally different from all EBPs, nor that implementation science has ignored complexity. Rather, we argue that certain features common to many contemporary AI systems, e.g., opacity, data dependence, probabilistic output, performance drift, vendor mediation and lifecycle instability, expose tensions with assumptions of stability, transparency and boundedness that are often implicit in implementation science frameworks. The aim of this paper is to examine how different types of AI systems align with, extend or challenge core assumptions in implementation science, using CFIR as a diagnostic lens and situating the analysis in relation to existing work on complex interventions and sociotechnical implementation.

## Method

This paper reports a comparative conceptual analysis rather than a systematic or scoping review. The purpose was not to exhaustively identify all studies on AI implementation, but to map recurring characteristics of AI implementation in conceptually relevant literature, and to examine where these characteristics may create conceptual tensions with assumptions embedded in implementation science frameworks developed primarily for EBPs.

The analysis drew on three bodies of literature. First, we examined foundational implementation science literature on EBPs, determinant frameworks, implementation processes, fidelity, adaptation, implementation outcomes and sustainment. Second, we examined conceptual and empirical literature on the implementation of AI in healthcare, including reviews of barriers and facilitators, studies of AI adoption, and papers addressing governance, trust, dataset shift, explainability and ethical oversight. Third, in order to conceptually align AI with other complex implementation objects, we examined literature on complex interventions and technology-enabled care, including Nonadoption, Abandonment, Scale-up, Spread and Sustainability (NASSS) [19], to avoid overstating the contrast between AI and other complex implementation objects.

Relevant literature was identified through an iterative purposive search strategy. Initial sources were identified from the authors’ prior work on implementation science and AI implementation. Additional sources were identified through citation tracking, reference chaining and targeted searches in PubMed, Google Scholar and Scopus using combinations of terms such as “artificial intelligence,” “implementation,” “healthcare,” “CFIR,” “barriers and facilitators,” “dataset shift,” “model drift,” “trust,” “explainability,” “governance,” “NASSS,” “complex interventions,” “fidelity,” and “adaptation.” We prioritized peer-reviewed reviews, conceptual papers, empirical studies and foundational implementation science texts that were directly relevant to the comparison.

Sources were included if they addressed: implementation of AI or digital health technologies in healthcare; theoretical assumptions in implementation science; implementation of EBPs; fidelity, adaptation or sustainment; sociotechnical complexity; or governance and evaluation of AI systems. Sources were excluded if they focused solely on technical model development without implications for implementation, or if they addressed AI in non-healthcare settings without transferable conceptual relevance.

The analysis proceeded in three steps. First, we identified assumptions commonly associated with implementation of EBPs in determinant and process frameworks, including relative stability of the intervention, specifiability of core components, pre-implementation evidentiary assessment, and distinction between intervention and context. Second, we identified recurring characteristics of AI implementation described in the literature, including opacity, data dependence, performance drift, infrastructure dependence, vendor entanglement, regulatory uncertainty, professional identity tensions and trust calibration. Third, we mapped these characteristics against CFIR’s five domains to identify where AI implementation could be accommodated within existing constructs and where it appeared to require theoretical refinement.

We used the updated CFIR [12] as the primary analytic lens. The updated CFIR retains CFIR’s multilevel structure while revising terminology and constructs, including replacing the original “Intervention Characteristics” domain with the broader “Innovation” domain. This change is important for the present analysis because AI systems are not always well captured by the term “intervention.” Some are clinical decision-support tools, some are infrastructural technologies, some are workflow systems, and some are general-purpose or foundation-model-based tools whose use may evolve after deployment. We therefore use “innovation” when referring to CFIR constructs, while retaining “intervention” when discussing the broader implementation science literature on EBPs and complex interventions.

CFIR organizes factors influencing implementation into five domains: the innovation, outer setting, inner setting, characteristics of individuals, and implementation process. Together, these domains capture how features of the implemented object, organizational context, wider policy and regulatory environments, individual users, and implementation activities shape adoption, use and sustainment [12].

CFIR was selected because it is one of the most widely used determinant frameworks in implementation science and because it explicitly organizes determinants across multiple levels. In this analysis, CFIR was used as an analytic and diagnostic lens rather than as a coding framework for empirical data. The aim was to examine conceptual fit, not to produce a complete taxonomy of determinants. This distinction is important: many AI-related implementation issues can be mapped onto CFIR domains, but mapping does not necessarily resolve whether the underlying assumptions of the framework are sufficient for dynamic, data-dependent and continuously governed systems.

## Results

The results identify systematic areas of overlap and tension between the implementation of EBPs and the implementation of AI across all five domains of the CFIR determinant framework [12].

### Innovation characteristics

#### Opacity

EBPs vary in the transparency of their mechanisms. Some are supported by clear causal theories and trial evidence, whereas others, particularly complex interventions, may work through multiple interacting mechanisms that are difficult to isolate [15]. Thus, opacity is not exclusive to AI. EBPs are often grounded in evidence such as clinical trials, systematic reviews, and professional guidelines. This allows users to examine mechanisms, assess causal reasoning, and evaluate the strength of the underlying evidence [10, 20, 21]. However, the degree to which mechanisms are transparent varies across EBPs, and implementation science has long recognized that intervention effects are shaped by context, adaptation and local enactment. This means that EBPs may provide an explicit rationale for action without necessarily making all mechanisms of effect fully observable or transferable across settings [15, 19, 20].

For many AI systems, particularly deep-learning and foundation-model-based systems, implementation involves a different form of opacity. The relationship between inputs, model parameters and outputs may be difficult to inspect or explain in clinically meaningful terms, even when aggregate performance is strong [22–24]. Their outputs are often correlations rather than explanations, raising concerns about trust, legitimacy, and accountability in high-stakes settings. This opacity is not merely a communication issue but is also intrinsic to many high-performing AI systems [22]. More precisely, many contemporary AI systems, particularly deep-learning and foundation-model-based systems, introduce a different form of opacity: the relationship between inputs, model parameters and outputs may be difficult to inspect or explain in clinically meaningful terms, even when aggregate performance is strong [22, 25, 26].

The extent of opacity differs by AI type. A locked rule-based decision support tool may be relatively transparent, while a deep-learning image classifier or generative AI system may be less inspectable. The implementation challenge is therefore not simply that users need more information, but that the system’s reasoning may not be fully explainable in clinically meaningful terms. Within CFIR, this can be mapped to complexity and evidence strength, but the issue also challenges the assumption that the implemented object can be sufficiently understood and appraised before adoption. This challenge is particularly salient where implementation depends on clinician, patient or organizational confidence in outputs whose internal logic cannot be readily reconstructed.

#### Performance drift

EBPs are not always stable interventions. Complex EBPs may be adapted to local context, and implementation science has long examined the tension between fidelity and adaptation [15, 16]. Nevertheless, many determinant and process frameworks assume that the core function of an intervention can be specified before implementation and that sustainment involves maintaining or adapting that function over time. EBPs are typically conceptualized as interventions whose effectiveness is established before implementation. Although fidelity and context influence outcomes, the core intervention is usually understood as identifiable and relatively stable [15, 16]. This should not be interpreted to mean that EBPs are static. Rather, many EBPs are introduced with a relatively specified evidentiary basis, intended user group and core function, even when local adaptation is expected [16, 27, 28].

For many AI systems, especially machine-learning systems deployed in changing clinical environments, data dependence creates vulnerability to dataset shift. Changes in populations, workflows, coding practices, or data quality can degrade performance, and model updates may alter outputs unpredictably. The intervention can therefore evolve after implementation [25, 26]. This issue is most pronounced for adaptive or periodically updated AI systems, but even locked systems may experience performance degradation if the deployment context changes. Generative AI systems add a further layer of instability because outputs may vary across similar prompts, users and contexts. This destabilizes the traditional trajectory from adoption to sustainment. Instead of moving toward stabilization alone, AI requires continuous monitoring, recalibration, validation, and, where necessary, de-implementation, shifting implementation toward ongoing lifecycle management rather than a time-limited change process [1, 29–27]. This claim is supported by methodological work on dataset shift and model degradation, as well as by emerging regulatory approaches that treat planned modification, post-market monitoring and lifecycle management as central to safe AI deployment [1, 25, 26, 29–31].

### Inner setting

#### Digital infrastructure as a precondition

Most EBPs do not typically depend on advanced data pipelines or substantial computational capacity, but have shown to require training, workflow redesign, and sustained leadership engagement [11, 32–28]. However, complex technology-enabled interventions may also depend heavily on infrastructure, interoperability and technical support. AI is therefore not unique in being infrastructurally dependent, but some AI systems make infrastructure constitutive of safe and effective functioning. A more precise distinction is that many EBPs require organizational infrastructure, whereas many AI systems additionally require computational, data and cybersecurity infrastructure. This distinction is not absolute: audit-and-feedback interventions, learning health systems and digital health interventions may also depend on data systems, interoperability and technical support [19, 33].

Implementation of many AI systems requires mature digital infrastructure: interoperable electronic health records, reliable and structured data pipelines, scalable computing resources, and robust cybersecurity safeguards. Some AI systems cannot function without these foundational elements [1]. For fixed AI systems, infrastructure may primarily concern integration, data access and workflow fit. For adaptive or data-dependent systems, infrastructure also includes continuous data pipelines, monitoring capacity and mechanisms for detecting drift. For generative AI, infrastructure also includes information governance, secure handling of clinical data, documentation of use and policies for human oversight. Infrastructure, therefore, is not only a contextual facilitator but may become a structural prerequisite. AI implementation shifts the focus from social and procedural change to a broader sociotechnical transformation. This transformation requires attention not only to technical readiness, but also to organizational responsibility for maintaining the data, interfaces, monitoring systems and governance processes on which safe AI use depends [2, 6, 7, 34].

#### Data as constitutive

For EBPs, data primarily support evaluation and monitoring. The intervention itself often functions independently of real-time data quality [17, 33]. This is not true for all EBPs; audit-and-feedback interventions, learning health systems and some digital interventions depend closely on data. Nevertheless, for many EBPs the data used to evaluate implementation are analytically separable from the intervention itself.

For many data-dependent AI systems, data are often constitutive of the intervention. For many machine-learning systems, data are not merely resources for evaluation but central to model development, local performance and post-deployment behavior. Incomplete, biased, or poorly structured data directly shape outputs. Data are not external inputs but integral components of the system’s behavior [6, 29]. This is particularly important for machine-learning systems trained on historical data and deployed in changing clinical contexts. A model trained on a defined dataset may be fixed at deployment, but its performance still depends on the relation between training data and local operational data. Empirical and methodological work on dataset shift shows that changes in populations, workflows, coding practices or data quality may alter model performance after deployment [25, 26]. Adaptive systems and generative systems further blur this boundary because their outputs may depend on continuously changing input data, prompts, retrieval systems or model updates.

Implementation science frameworks typically distinguish between the intervention and its context, treating factors such as “evidence strength” and “organizational readiness” as determinants [5]. AI disrupts this separation when the intervention is inseparable from a continuously evolving data ecosystem. Building on the dataset-shift and AI-lifecycle literature, we propose that, for data-dependent AI systems, the local data ecosystem should be treated as partly constitutive of the implemented innovation rather than merely as contextual background [1, 29, 34].

#### Cross-disciplinary coordination

Implementation of EBP often requires coordination among clinicians and managers [11, 18]. Complex interventions may also require collaboration among multiple professional groups, service sectors and organizational levels. Implementation of many AI systems, however, often demands sustained collaboration among clinicians, data scientists, IT professionals, legal experts, compliance officers, procurement specialists, information governance staff and executives. Technical robustness, regulatory alignment, workflow integration, and ethical oversight must be managed simultaneously [1, 35]. This does not mean that AI implementation is always more complex than all EBP implementation. Rather, it means that AI often combines clinical, technical, ethical, legal and commercial dependencies in ways that are difficult to manage through conventional clinical implementation structures alone. Implementation of AI is not merely a professional practice change; it involves orchestrating technical, regulatory, ethical, and operational systems in parallel [7, 29]. The coordination challenge is therefore sociotechnical: successful implementation depends on aligning clinical work, data infrastructure, governance authority, vendor arrangements and user practices rather than simply installing a tool or training end users [1, 19, 34].

### Outer setting

#### Regulatory ambiguity

EBPs are generally embedded within established professional guidelines, accreditation systems, and regulatory frameworks, reducing uncertainty regarding compliance and legitimacy and creating a relatively predictable external implementation environment [11, 36, 37]. However, EBPs can also be implemented amid changing recommendations, contested evidence and shifting policy environments. The contrast is therefore not absolute. Many EBPs are implemented within relatively established professional, organizational and policy environments, including clinical guidelines, accreditation systems, quality standards and professional norms [10, 20, 36, 37]. However, the evidentiary and regulatory environment for EBPs can also change over time, especially when new evidence, safety concerns or policy priorities emerge.

Many AI systems, particularly AI-enabled medical devices and clinical decision-support systems, operate within evolving regulatory landscapes, shifting liability standards, and ambiguous accountability structures. Questions such as who is responsible when an algorithm errs remain unsettled in many jurisdictions [1, 29]. Regulatory uncertainty increases organizational risk aversion and hesitancy [2, 6]. The implications differ by AI type. A fixed, pre-approved AI tool with a narrow intended use may fit more readily into existing regulatory categories. Adaptive and generative systems raise more difficult questions because model updates, broad use cases and variable outputs may complicate approval, monitoring and accountability. Thus, external uncertainty is structurally higher for some AI systems than for many EBPs. More specifically, regulatory uncertainty appears particularly salient for AI-enabled medical devices and clinical decision-support systems because regulatory approaches must address software updates, changing model performance, liability, human oversight and post-market monitoring [30, 31, 38]. Recent guidance on predetermined change control plans illustrates this lifecycle orientation by addressing planned modifications to AI-enabled device software and continued safety and effectiveness across modifications [31].

#### Ethical concerns

Many AI systems raise heightened ethical concerns related to bias, fairness, and discrimination. Empirical evidence shows that algorithms can reproduce or amplify inequities embedded in historical data [23]. Although EBPs also raise ethical considerations, these are typically addressed during research, peer review, and development of guidelines [10, 20]. This does not mean that EBPs are ethically settled once implemented; inequitable uptake, differential access, cultural mismatch and unintended consequences are well-recognized implementation issues. Therefore, the ethical distinction between EBPs and AI should not be framed as the presence versus absence of ethical concern. EBPs also raise ethical issues, including inequitable access, differential uptake, cultural mismatch, implementation burden and unintended consequences. These concerns are addressed through research ethics, peer review, guideline development, professional standards and implementation monitoring [17, 39].

Some AI systems introduce ongoing ethical risk due to dynamic data inputs, performance drift and subgroup variation. Ethical oversight must therefore be continuous rather than confined to pre-implementation review [26–29]. For generative AI, additional ethical concerns include hallucination, inappropriate disclosure of sensitive information, unclear authorship, and the possibility that outputs may appear authoritative despite being inaccurate or unsupported. These concerns can be mapped to existing constructs such as evidence strength, patient needs and external policy, but they also require implementation frameworks to account for continuous ethical monitoring as part of routine operation. We therefore treat continuous ethical oversight for AI not as an entirely new ethical category, but as an extension and intensification of existing concerns about equity, safety and accountability in implementation [23, 26, 29].

#### Vendor dependence

Many AI systems are proprietary, limiting transparency and independent evaluation. Organizations may become dependent on vendors for updates, maintenance, and performance monitoring [1, 19]. By comparison, EBPs are less often proprietary technologies in the same sense, and organizations may retain greater autonomy over adaptation and sustainment [11, 18]. However, vendor dependence is not unique to AI; many digital health technologies, electronic health records and medical devices involve commercial suppliers, contractual constraints and interoperability challenges. Vendor dependence is therefore best understood as a broader issue in digital and technology-enabled care, rather than as a feature unique to AI [19, 34].

The distinctive issue for some AI systems is that vendor dependence may shape the behavior of the implemented intervention itself. If model updates, training data, performance monitoring, interface design or explainability tools are controlled by vendors, then organizations may have limited ability to inspect, adapt or govern the system independently.

Vendor entanglement introduces commercial and contractual dynamics that reshape power relations in implementation [40]. Within CFIR, vendor dependence can be mapped to the outer setting or intervention characteristics, but for AI it may also be constitutive of the intervention lifecycle. Building on existing work on digital health implementation and algorithmic management, we propose that vendor dependence may be especially consequential for adaptive and generative AI when vendors control model updates, monitoring tools, training data, interface changes or explainability functions [1, 29, 40]. In such cases, vendor governance is not only an outer-setting determinant but part of lifecycle stewardship.

### Characteristics of individuals

#### Technical literacy

Implementing EBPs typically requires clinical knowledge and procedural training aligned with existing professional competencies [10–11, 18]. Many EBPs and complex interventions also require new skills, new forms of teamwork and changes in professional routines. AI systems additionally require familiarity with probabilistic outputs, algorithmic reasoning, uncertainty, data limitations and appropriate human oversight [24]. Poorly designed systems can contribute to cognitive overload, undermining usability and appropriate reliance [2, 41].

The level of required technical literacy differs across AI systems. A locked AI alert may require users to understand sensitivity, specificity, false positives and false negatives. A predictive risk model may require interpretation of calibration and local validation. A generative AI system may require awareness of hallucination, prompt sensitivity, uncertainty, and the need to verify outputs against clinical evidence and patient context. Thus, implementation of AI often demands forms of technical literacy beyond understanding of the scientific principles included in the professional education of most healthcare leadership and staff. This does not imply that all clinicians must become data scientists. Rather, AI implementation requires role-appropriate literacy: end users need sufficient understanding to interpret outputs and limitations, while implementation leaders require enough technical and governance literacy to make decisions about procurement, validation, monitoring and accountability.

#### Professional identity

EBPs frequently reinforce professional identity by drawing on disciplinary evidence traditions [10, 42, 43]. However, EBPs can also challenge professional autonomy when they standardize practice, alter discretion or redistribute tasks. Implementation science has therefore long recognized professional identity and role negotiation as relevant to implementation. Some AI systems may further professional autonomy through its capacity to present rival epistemological claims based on large-scale data patterns or model-generated outputs. Some clinicians perceive AI as a threat to expertise or decision-making authority. Concerns about deskilling, role displacement, and shifting professional boundaries shape resistance and ambivalence [44–47]. A more nuanced interpretation is that EBPs and AI can both redistribute authority, but they do so in different ways. EBPs may privilege guideline-based or trial-based evidence over individual discretion, whereas AI may introduce algorithmically generated outputs that appear to compete with professional judgement in real time [42–45].

The theoretical issue is not merely that AI creates resistance. Rather, AI may introduce a new epistemic actor into clinical work: a system whose recommendations may appear to compete with professional judgement, guidelines or patient preferences. This issue is especially salient for generative AI and decision-support systems that provide recommendations, summaries or interpretations. Identity tensions might therefore be more pronounced in the implementation of some AI systems than with many EBPs, although they are not entirely unique to AI. We interpret these tensions as especially salient where AI systems provide recommendations, summaries, classifications or interpretations rather than merely automating narrow technical tasks [46, 47].

#### Trust calibration

Because EBPs are grounded in research evidence and embedded within professional norms, trust often develops through familiarity with the evidence base and experiential use [10, 18]. However, trust in EBPs is also variable and can be shaped by professional values, patient preferences, local experience and confidence in the producing evidence institutions. Many AI systems require calibrated trust, because implementation may fail through both overreliance and underutilization. Trust must be actively cultivated through transparency mechanisms, training, performance monitoring, and governance [23, 24, 48]. Trust has been identified empirically as a recurring issue in AI implementation and adoption [48, 49]. The concept of calibrated trust is also well established in human–automation research, where the central concern is to avoid both misuse and disuse of automated systems [50, 51].

This is especially important because inappropriate reliance may take two forms: automation bias, where users accept AI outputs too readily, and algorithm aversion, where users reject useful AI outputs despite evidence of performance. For adaptive or generative AI systems, trust must also be periodically recalibrated as performance, context or use cases change. Trust in AI is therefore not a stable attitudinal determinant only; it is a dynamic relation among user understanding, local performance evidence, transparency, accountability and governance [26–29, 49]. In this paper, we extend the idea of calibrated trust to implementation science by proposing that trust in adaptive or generative AI should be treated as dynamic and periodically recalibrated as local performance, use cases and governance arrangements change.

### Implementation process

#### Continuous monitoring

Sustainment of EBPs focuses on ongoing fidelity and outcomes [17, 52]. However, sustainment research also recognizes adaptation, fit, changing context and the need to maintain intervention benefit over time. The distinction between implementation and sustainment is therefore not always clear-cut. Many AI systems require post-deployment performance monitoring to detect drift, assess subgroup impacts and guide recalibration. For adaptive, data-dependent and generative systems, this monitoring may need to be continuous or periodically repeated. The intervention may never be fully stabilized in the conventional sense. This alters the temporal logic of implementation. For EBPs, monitoring is often oriented toward maintaining fidelity, assessing outcomes and supporting sustainment. For AI systems, monitoring must also evaluate whether the technical behavior of the system remains safe, valid and equitable under changing data and workflow conditions.

For fixed AI systems, monitoring may focus on whether local performance remains consistent with pre-implementation evidence. For adaptive systems, monitoring must also address the consequences of updates, recalibration and changing data environments. For generative AI, monitoring must include the appropriateness, safety and reliability of outputs across a potentially broad and evolving set of use cases. Adoption is not a discrete event but the beginning of ongoing technical stewardship [25, 26]. For adaptive, data-dependent and generative AI systems, adoption is better understood as the beginning of ongoing technical and organizational stewardship. This claim is supported by methodological work on dataset shift and post-deployment performance degradation, by AI evaluation and reporting guidance emphasizing clinical evaluation of AI systems in use, and by regulatory approaches that increasingly emphasize lifecycle management and change control for AI-enabled medical devices [25, 26, 30, 31, 53–55].

#### Iterative validation

Pilot testing for EBPs typically assesses feasibility, acceptability, and appropriateness in terms of provider burden, workflow integration, perceived relevance to client needs, and preliminary signals of implementation success [17, 39]. For complex interventions, pilots may also examine mechanisms, context sensitivity and adaptations. Piloting of many AI systems should additionally evaluate local performance, subgroup performance, bias distribution, calibration, robustness and fairness across demographic groups and clinical contexts. Responsible deployment requires stress testing across contexts and data environments [23].

The key point is not that AI alone requires evaluation, but that validation cannot be confined to pre-implementation evidence. A model developed and validated in one dataset may perform differently in another organization because of differences in populations, workflows, coding practices or data quality. Generative AI systems further require evaluation of output quality, hallucination risk, information leakage, consistency and appropriateness for specific tasks. Piloting, therefore, expands beyond traditional feasibility, acceptability, and appropriateness into iterative technical and ethical validation [1]. Reporting and evaluation guidance for AI interventions reinforces this point by emphasizing the need to describe the AI system, input data, human–AI interaction, intended use, errors, safety issues and clinical context [53–55]. Thus, iterative validation should be understood as both a technical requirement and an implementation process: it links local evidence generation, workflow fit, equity assessment, user trust and governance decisions.

#### Governance and lifecycle management

Many AI systems, particularly adaptive, data-dependent, proprietary or clinically consequential systems, require formal governance structures to oversee updates, accountability, transparency, and vendor relationships [29, 35]. EBPs generally do not require comparable technical lifecycle management; governance primarily ensures fidelity, adaptation, quality improvement and accountability [16, 17]. However, governance is also important for EBPs, particularly when interventions are complex, high-risk or implemented across organizations. The difference is that for some AI systems, governance is not merely a support for implementation but part of the implemented object itself. This distinction should be framed as a matter of degree and function rather than presence or absence: both EBPs and AI require governance, but adaptive, data-dependent and proprietary AI systems may require governance mechanisms that directly shape the system’s continuing behavior.

The implemented AI system includes the algorithm, data pipeline, user interface, workflow, monitoring processes, update mechanisms, human oversight, vendor arrangements and accountability structures. Accordingly, implementation of AI is best understood not only as a bounded rollout but as an enduring management and oversight process [19, 25]. This is particularly true for adaptive and generative systems, where changes in model behavior, use context or user practices may alter the risk-benefit profile after deployment. We use the term lifecycle stewardship as a conceptual synthesis of the dataset-shift, AI evaluation, sociotechnical implementation and regulatory literatures, rather than as an already established implementation model [1, 25, 26, 29, 31, 34, 53]. This concept emphasizes that AI implementation requires ongoing responsibility for model performance, data quality, safety, equity, accountability, vendor relations and de-implementation when benefits no longer outweigh risks.

## Discussion

The analysis shows that AI should not be treated as a single implementation object. Some AI systems, particularly fixed or locked tools with a narrow intended use, may be implemented in ways that resemble other complex digital interventions. However, adaptive, data-dependent, opaque and generative AI systems create stronger tensions with assumptions that are often implicit in implementation science frameworks: that interventions can be specified before implementation, that their evidentiary basis can be established before rollout, that intervention and context can be analytically separated, and that implementation moves toward stabilization over time.

Not all AI-related implementation issues require new theory. Many can be accommodated within existing CFIR domains. For example, regulatory uncertainty can be mapped to the outer setting, and technical literacy can be mapped to characteristics of individuals. The conceptual issue is not whether CFIR can classify these factors, but whether classification alone captures their role in AI implementation. For some AI systems, data quality, vendor-controlled updates and monitoring arrangements do not merely influence implementation; they partly constitute the intervention itself. Similarly, performance drift is not only a barrier to sustainment but a feature that changes the object being sustained. The most important theoretical tensions therefore concern the stability of the intervention, the boundary between intervention and context, and the temporal distinction between implementation and sustainment.

Within organizations, implementation of AI is not primarily a matter of training and workflow alignment. It requires durable digital infrastructure, high-quality data ecosystems, cybersecurity capacity, and formal governance mechanisms. These are structural conditions, not facilitative add-ons. If AI is approached as a conventional practice change within implementation science, there is a risk of underestimating its infrastructural fragility and overestimating organizational readiness. The result may be premature deployment, hidden technical debt, and institutional dependence on external vendors.

In the outer setting, AI operates within evolving regulatory regimes and unsettled liability standards, intensifying ethical consideration. Bias, discrimination, and accountability are not one-time review issues but ongoing risks amplified by continuous learning and dataset shift. Conceptualizing AI as a discrete innovation may compress these systemic uncertainties into conventional “outer setting” determinants, thereby minimizing their structural significance. This mischaracterization can produce governance gaps precisely where vigilance is most needed.

At the individual level, AI requires new forms of engagement with knowledge, including probabilistic reasoning, interpretation of uncertainty, and calibrated trust. It may also reconfigure professional identity and authority. If implementation frameworks assume alignment with existing professional norms, they risk overlooking identity threats, overreliance, automation bias, algorithm aversion and erosion of skills. These are not marginal implementation barriers; they are shifts in the cognitive and moral architecture of practice.

AI also disrupts the temporal logic of implementation. Implementation science has often been organized around phases such as exploration, adoption, implementation and sustainment. These phases remain useful, but for adaptive and data-dependent AI systems they may not adequately capture the ongoing nature of monitoring, recalibration, validation and governance. Deployment is not the endpoint of evaluation but the beginning of continuous surveillance, recalibration, and oversight. For some AI systems, there may be no stable “post-implementation” state in the conventional sense. Treating AI as a time-limited change effort risks substituting episodic evaluation for permanent stewardship. Resolving issues in these areas of concern will likely demand interventions that target barriers and facilitators not hitherto identified in taxonomies of implementation strategies in the field of implementation science. Organizational attention will, for example, need to be directed at facilitating negotiations around AI as a new epistemic knowledge source in the workplace.

Our argument should be read alongside, rather than in opposition to, existing work on complex interventions, sociotechnical systems and NASSS. We do not claim that implementation science uniformly treats interventions as fixed or context-free. On the contrary, substantial work has examined adaptation, fidelity, intervention function versus form, sustainment, de-implementation and the embedding of interventions in complex systems [15–19, 27, 52]. These strands of scholarship already problematize simple assumptions of linear implementation. AI should therefore be understood as an intensifying case rather than an entirely unprecedented case. It sharpens existing theoretical questions because the intervention’s behavior may change through data shifts, model updates and vendor decisions, not only through human adaptation or local tailoring.

NASSS already addresses many issues that are highly relevant to AI implementation, including technological complexity, organizational readiness, value propositions, adopter work, wider institutional conditions and change over time [19]. It therefore offers a more suitable starting point for AI implementation than frameworks that assume relatively bounded interventions and linear implementation trajectories.

However, our analysis adds to NASSS in three ways. First, by using CFIR diagnostically, we show how determinant frameworks commonly applied in implementation research may absorb AI-related issues into existing categories without questioning whether the underlying categories remain sufficient. Second, we specify AI-related phenomena that require further theoretical elaboration: model drift, dataset shift, algorithmic opacity, probabilistic outputs, vendor-mediated updating, data ecosystem dependence, and continuous ethical oversight. Third, we argue that for some AI systems, governance is not merely a contextual condition influencing implementation but a constitutive element of the intervention itself. The implemented object is not only a technology but an evolving sociotechnical arrangement consisting of model, data pipeline, workflow, user practices, vendor relationship, monitoring procedures and accountability mechanisms. Thus, the contribution of this paper is not to claim that NASSS is inadequate, but to specify how implementation science frameworks, including but not limited to CFIR and NASSS, might be extended when the object of implementation is dynamic, data-dependent and continuously governed.

Many implementation challenges associated with AI are not unique to AI. Digital health technologies, electronic health records, telemedicine platforms and complex service innovations also raise issues of workflow disruption, infrastructure dependence, user burden, vendor relationships and sustainability. The distinctiveness of AI is therefore best understood configurationally rather than categorically. AI becomes theoretically challenging when several features coincide: probabilistic and opaque outputs, dependence on local data distributions, susceptibility to performance drift, vendor-controlled updates, regulatory ambiguity and the need for continuous monitoring. These features are not equally present in all AI systems, but when they co-occur they stretch implementation frameworks beyond conventional determinant mapping.

This means that the relevant question is not whether AI is always “same-same” or always fundamentally different. Rather, the question is under what conditions an AI system becomes a distinctive implementation object. A locked model with a narrow use case, stable input data and clear regulatory approval may be implemented using existing frameworks with modest extension. Conversely, an adaptive or generative system with broad use cases, uncertain accountability, vendor-controlled updates and changing data inputs requires a stronger shift toward lifecycle stewardship, continuous validation and governance.

Based on the preceding analysis, we propose that implementation science frameworks require refinement when applied to AI in several interrelated ways. AI implementation should be viewed as lifecycle stewardship rather than discrete rollout, requiring ongoing monitoring, validation, updating, recalibration and possible de-implementation. The implemented object should be understood as a sociotechnical assemblage that includes the algorithm, data pipeline, interface, workflow integration, human oversight, vendor relationships, monitoring procedures and governance arrangements. Data ecosystems should be treated as constitutive of implementation, as data quality, representativeness, interoperability and coding practices shape system behavior. Trust should be conceptualized as calibrated and dynamic, distinguishing appropriate reliance from overreliance and underuse. Finally, vendor and regulatory governance should be central analytic domains, since proprietary systems, contractual dependencies, liability arrangements and update control determine what can be known, modified and monitored.

Lifecycle evaluation of AI should not collapse all relevant indicators into “implementation outcomes.” The CFIR Outcomes Addendum [56] helps clarify this by distinguishing implementation outcomes, such as acceptability, adoption, appropriateness, feasibility, fidelity, cost, penetration and sustainability, from outcomes related to the innovation itself, service delivery, patients, effectiveness, safety and equity. For AI systems, measures such as calibration, discrimination, subgroup performance, fairness, drift, hallucination frequency and output reliability are better understood as indicators of innovation performance, safety or equity, rather than implementation outcomes in the narrow sense.

This distinction matters because implementation conditions and AI performance may shape each other over time. Poor workflow integration may degrade data quality and calibration; limited clinician trust may reduce adoption despite strong model performance; automation bias may increase use while undermining safety; and subgroup performance problems may require adaptation, retraining, governance intervention or de-implementation. Lifecycle evaluation should therefore monitor implementation outcomes alongside AI performance, clinical effectiveness, safety, equity, service and patient outcomes. This preserves conceptual clarity while recognizing that AI implementation cannot be judged by adoption or sustainment alone: systems may be widely used yet drift or produce inequitable effects, while technically strong systems may fail if they are unacceptable, infeasible, poorly integrated or insufficiently trusted.

This paper should be situated alongside previous efforts to identify determinants of the implementation of AI and describe stages of activities to achieve successful implementation. Two reviews have explicitly applied CFIR to the implementation of AI [6, 7]. Although they offer valuable multilevel insights, they largely treat AI as an innovation to be mapped onto existing implementation categories, i.e., CFIR domains [11], rather than questioning whether those categories, developed for implementation of EBPs, remain conceptually sufficient. In contrast, this paper uses CFIR [11] comparatively and diagnostically to examine whether its underlying assumptions hold when the implementation objects are dynamic, data dependent, and continuously evolving AI systems.

Other reviews have cataloged determinants of implementation of AI using alternative frameworks [2, 3, 8], 57– 34]. These analyses similarly focus on identifying determinants of implementation of AI rather than examining whether those determinants differ in kind from those characterizing implementation of EBPs. Our revised argument is not that such reviews are incorrect, but that determinant mapping alone may be insufficient if AI-related issues such as drift, data dependence and vendor-controlled updating are treated only as barriers or facilitators rather than as features of the implemented object. The distinctive contribution of this paper is its shift from determinant mapping to questioning theoretical adequacy.

Attempts have also been made to adapt process models for implementation of EBPs [5], such as the QIF [59], to guide implementation of AI systems [9]. These adaptations implicitly assume that the core stages and activities developed for implementation of EBPs are transferable to AI, and that AI systems can be translated into practice through broadly similar procedural logics. This may be reasonable for some fixed AI tools, but it is less clear for adaptive, data-dependent or generative AI systems whose performance and risk profile may change after deployment. Whether this assumption is empirically warranted or conceptually defensible therefore requires careful examination.

This paper has several limitations. It does not constitute a systematic or scoping review of research on implementation of AI. Rather than providing an exhaustive synthesis of the empirical literature, the analysis selectively engages influential conceptual, empirical, and review studies to identify recurring structural characteristics of implementation of AI. It should therefore be understood as a theoretically informed comparative analysis rather than a comprehensive evidence synthesis. Alternative literature selections or theoretical lenses might generate additional insights or refine the contrasts presented here.

A further limitation is that AI itself is a rapidly evolving category. The distinctions made here between fixed, adaptive and generative AI systems are analytically useful, but real-world systems may combine features of several types. For example, a fixed predictive model may be embedded in a changing software interface, while a generative AI system may be constrained by local governance rules or retrieval systems. The categories should therefore be understood as heuristic rather than definitive.

A third limitation is that our comparison necessarily simplifies both AI and EBP implementation. EBPs can be complex, contested, adapted and dependent on organizational systems, and many concerns raised here have parallels in implementation of digital health technologies and other sociotechnical interventions. We have therefore attempted to avoid a binary contrast between stable EBPs and unstable AI systems. Nonetheless, further empirical research is needed to examine which AI features create distinctive implementation challenges in specific settings.

Finally, CFIR was used as the primary analytic lens because of its prominence in implementation science and its prior use in AI implementation reviews. Other frameworks, particularly NASSS, normalization process theory, complexity-informed approaches and learning health system models, may foreground different issues. Future work should compare how different frameworks conceptualize AI implementation and examine whether integrated or AI-specific frameworks offer additional explanatory value.

## Conclusion

AI is not inherently “just another innovation,” but neither is it categorically separate from other complex interventions. Its implementation implications depend on the type of AI system and the extent to which it is fixed, adaptive, data-dependent, opaque, generative, vendor-mediated or clinically consequential. Existing implementation science frameworks such as CFIR and NASSS remain valuable for identifying multilevel determinants, but they require careful extension when the implemented object is dynamic, sociotechnical and continuously governed. For such systems, implementation should be understood less as a bounded rollout and more as lifecycle stewardship involving local validation, monitoring of performance and equity, calibrated trust, governance of data and vendors, and readiness for adaptation or de-implementation. AI therefore does not render implementation science obsolete; it exposes where its assumptions about stability, boundedness and evidentiary closure needs to evolve.

## Data Availability

No datasets were generated or analysed during the current study.

## Abbreviations

AI: Artificial Intelligence
EBP: Evidence-based practice
CFIR: Consolidated Framework for Implementation Research
QIF: Quality Implementation Framework
NASSS: Nonadoption, Abandonment, Scale-up, Spread and Sustainability
